# Real-Time Detection of Yeast Growth on Solid Medium through Passive Microresonator Biosensor

**DOI:** 10.3390/bios14050216

**Published:** 2024-04-26

**Authors:** Bo-Wen Shi, Jun-Ming Zhao, Yi-Ke Wang, Yan-Xiong Wang, Yan-Feng Jiang, Gang-Long Yang, Jicheng Wang, Tian Qiang

**Affiliations:** 1 School of Internet of Things Engineering, Institute of Advanced Technology, Jiangnan University, Wuxi 214122, China; 1038210214@stu.jiangnan.edu.cn (B.-W.S.); 1038210218@stu.jiangnan.edu.cn (J.-M.Z.); 1038210220@stu.jiangnan.edu.cn (Y.-K.W.); 7231923001@stu.jiangnan.edu.cn (Y.-X.W.); jiangyf@jiangnan.edu.cn (Y.-F.J.); 2 State Key Laboratory of Biochemical Engineering, Institute of Process Engineering, Chinese Academy of Sciences, Beijing 100190, China; glyang@ipe.ac.cn; 3 Key Laboratory of Biopharmaceutical Preparation and Delivery, Chinese Academy of Sciences, Beijing 100190, China; 4 School of Science, Jiangsu Provincial Research Center of Light Industrial Optoelectronic Engineering and Technology, Jiangnan University, Wuxi 214122, China; jcwang@jiangnan.edu.cn

**Keywords:** integrated passive device, biosensor, yeast, real-time biomonitoring, capacitive sensor

## Abstract

This study presents a biosensor fabricated based on integrated passive device (IPD) technology to measure microbial growth on solid media in real-time. Yeast (*Pichia pastoris*, strain GS115) is used as a model organism to demonstrate biosensor performance. The biosensor comprises an interdigital capacitor in the center with a helical inductive structure surrounding it. Additionally, 12 air bridges are added to the capacitor to increase the strength of the electric field radiated by the biosensor at the same height. Feasibility is verified by using a capacitive biosensor, and the change in capacitance values during the capacitance detection process with the growth of yeast indicates that the growth of yeast can induce changes in electrical parameters. The proposed IPD-based biosensor is used to measure yeast drop-added on a 3 mm medium for 100 h at an operating frequency of 1.84 GHz. The resonant amplitude of the biosensor varies continuously from 24 to 72 h due to the change in colony height during vertical growth of the yeast, with a maximum change of 0.21 dB. The overall measurement results also fit well with the Gompertz curve. The change in resonant amplitude between 24 and 72 h is then analyzed and reveals a linear relationship with time with a coefficient of determination of 0.9844, indicating that the biosensor is suitable for monitoring yeast growth. Thus, the proposed biosensor is proved to have potential in the field of microbial proliferation detection.

## 1. Introduction

Microorganisms have a wide range of applications in human life. Take yeast for example, which is commonly seen in daily life; within the realm of food fermentation, yeast’s enzymatic activities are pivotal, encompassing starch hydrolysis, dough leavening, alcohol production, and metabolite synthesis [1]. In the field of animal husbandry, yeast hydrolysate, rich in nucleotides and amino acids, is crucial for biosynthesis and metabolic processes in animals, thereby exerting a notable enhancement on growth [2]. Among these, *Pichia pastoris* GS115 strain, in particular, is considered one of the most popular strains used as an important expression system in industrial and medicine fields [3]. Considering these applications, detection of yeast as well as other microbial growth has become necessary in recent decades.

Real-time microbial proliferation is often used to demonstrate biosensor performance. As for yeast, most traditional methods for monitoring biological parameters during yeast growth are performed offline. These methods involve measurement errors, require a significant amount of time and labor costs, and fail to obtain morphological information about yeast growth. Therefore, it is necessary to develop new methods for online monitoring of yeast growth [4]. The optical density technique is commonly utilized for real-time biomass assessment, yet its accuracy can be compromised by any substance in the culture medium that alters turbidity [5]. Raman spectroscopy has gained traction for its optical analysis capabilities in yeast fermentation monitoring [6,7], and its precision is further enhanced when paired with sophisticated algorithms [8]. Additionally, in situ microscopy has been adopted for tracking yeast proliferation [9,10]. This noninvasive technique allows for the direct acquisition and visualization of microscopic images from the culture, offering a window into the yeast’s growth environment. In addition, in the current field of thermodynamics as well as mechanical resonators, various methods are being used to detect yeast growth [11]. In the realm of electronics, electrical biosensors have been deployed for biological detection applications [12]. Specifically in the context of yeast detection, key electrical parameters, including capacitance and impedance, are instrumental in delineating a multitude of characteristics pertaining to the fermentation broth [5,13,14]. Geng devised a microfluidic electrode array tailored for the electrical impedance spectroscopy of individual yeast cells [15]. Currently, the monitoring of yeast growth based on electrical biosensors is mainly based on liquid media, with limited research on yeast growth monitoring on solid media.

Microwave frequency band biosensors are indeed garnering significant interest in the field of detection due to their high sensitivity, real-time measurement capabilities, fast response times, robustness, label-free detection, and cost-effectiveness [16,17,18]. These biosensors have been effectively utilized in chemical detection, notably in identifying substances like ethanol [19,20,21,22] and various gases [23,24,25,26]. In the biological domain, microwave biosensors have demonstrated proficiency in sample detection. For instance, Tamra introduced a 40 GHz microwave biosensor designed for monitoring and characterizing single cells (THP-1) during electrochemotherapy, capturing an electronic signature indicative of treatment efficacy [27]. Kim’s contribution to the field includes a novel terahertz (THz) Fabry–Perot (FP) microcavity biosensor, employing a porous polytetrafluoroethylene (PTFE) film to enhance microorganism detection [28]. Despite these advancements, the application of microwave biosensors for real-time microbial growth monitoring on solid media remains less explored compared to their use for solution concentration and cell count assessments. Mohammadi’s work, which features a new label-free microwave biosensor based on planar split ring resonators, marks a step forward in the noninvasive monitoring of bacterial growth on solid agar media [29]. However, relatively few studies have been conducted on real-time monitoring of yeast on solid media using microwave biosensors. Unlike liquid media, solid media have important applications in the isolation, identification, and preservation of microorganisms. Therefore, a biosensor that can detect the growth of yeast on solid media in real-time appears to be very necessary in daily applications.

In this study, we propose a microwave biosensor used to measure the change in electrical signals during microbial growth, and we use the biosensor to detect yeast proliferation to demonstrate its potential in microbial detection. We initially establish the feasibility of this study via a capacitive biosensor. Subsequently, we develop a microwave microresonator-based biosensor designed for the real-time monitoring of yeast growth on solid media. The biosensor is manufactured using the IPD process on a GaAs substrate and consists of a circular spiral inductor and an interdigital capacitor at the center position. These components can sensitively detect changes in electrical signals in the environment. Throughout the growth cycle, the contact area of the yeast colony with the medium remains unchanged; however, the colony’s height increases. This growth alters the biosensor’s resonant amplitude. Notably, the resonant amplitude exhibits a linear correlation with time during the yeast growth phase. This relationship facilitates the use of peripheral circuits to represent the state of yeast growth in further investigations. This work demonstrates the potential that microwave biosensors have for microbial proliferation detection in real-time.

## 2. Biosensor Design and Analysis

**Figure 1:**
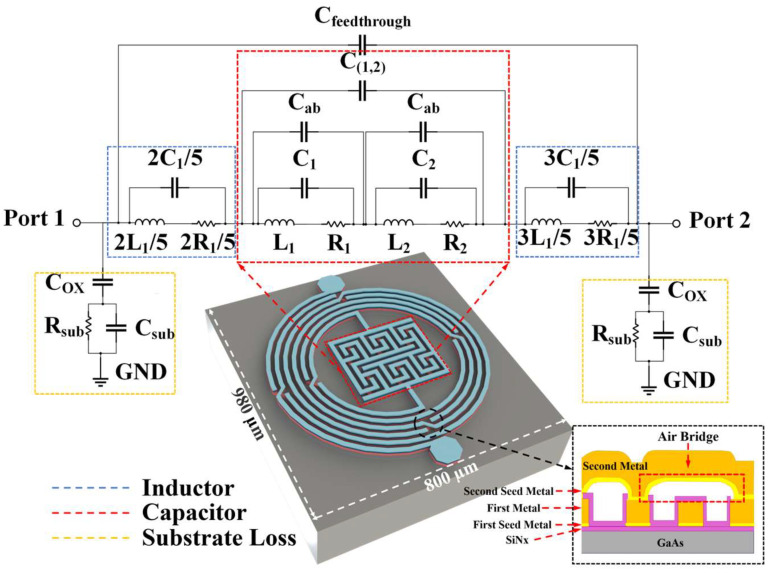
3D structure of the proposed biosensor with its equivalent circuit model and structure of air bridge section.

Figure 1 shows the 3D structure of the proposed biosensor as well as its equivalent circuit model. As shown in the inset image from bottom to top, it consists of a 200 μm GaAs substrate, a 0.2 μm SiNx passivation layer, a 20/80 nm Ti/Au first seed metal layer, a 4.5/0.5 µm Cu/Au first metal layer, a 0.2 μm SiNx sidewall protection layer, a 20/80 nm Ti/Au second seed metal layer, and a 4.5/0.5 µm Cu/Au second metal layer. An air bridge structure with a height of 1.8 μm is formed between the first metal layer and second metal layer. Seed metal Ti/Au is used for the electroplating process of the first and second metal layer, so that a thick metal with a height of 5 µm could be obtained, which leads to an excellent electric performance with a higher quality factor and lower loss for the inductor and capacitor [30]. Furthermore, the SiNx layer is applied to protect the sidewall of copper metal from environmental oxidation, making the fabricated biosensor chip with high reliability [31]. As depicted in Figure 1, the proposed biosensor, leveraging integrated passive device (IPD) technology, incorporates an asymmetrical spiral inductor and a refined interdigital capacitor. The biosensor’s compact design occupies a minimal area of 800 µm × 980 µm. The inductor is meticulously crafted with 5-turn intertwined meander lines, each line boasting a 15 µm width and 15 µm spacing. The structure’s dimensions are precisely defined with an inner diameter of 275 µm and an outer diameter of 425 µm, optimizing the biosensor’s performance. In order to promote the Q-factor and ensure connection at the same time [30], five air bridges are placed in every circle of the inductor, shown in Figure 1 as well.

The whole inductor can be approximated as a circular inductor, which is determined as follows [32]:

(1) $$L=6.28n^{2}D_{\mathrm{av}}\left(20\rho^{2}-100\ln\rho +90.02\right)\;(nH).$$

In the formula, n is used to represent the number of the circles, while ρ and D_av_, respectively, stand for the fill ratio and the average diameter of the inductor. Both of them can be obtained using the following equations:

(2) $$\rho =\frac{D_{o}-D_{i}}{D_{o}+D_{i}},$$

(3) $$D_{\mathrm{av}}=\frac{1}{2}\left(D_{o}+D_{i}\right).$$

D_o_ stands for the outside diameter of the inductor, and D_i_ denotes the inside diameter of the inductor. 

The configuration of the capacitors involves two interdigital capacitors placed in close proximity to each other, serving as the principal capacitance elements of the device. To further enhance the device’s sensitivity, the design incorporates 12 air bridges within these interdigital capacitors. These air bridges expand the sensitive area of the capacitors, thereby boosting their overall capacitance and sensitivity. In Figure 1, C_ab_ is used to stand for the capacitance of air bridges inside the capacitor section. At the same time, C_(1,2)_ means the capacitance among the two interdigital capacitors. As for the substrate loss, the biosensor generates various parasitic effects at high frequencies owing to its multilayer structure. C_ox_ is the parasitic capacitance formed between the planar pattern and the lossy substrate and can be calculated as follows: [33]

(4) $${\;C}_{\mathrm{ox}}=\frac{1}{2}\mathrm{lw}\frac{\varepsilon_{\mathrm{SiN}x}}{t_{\mathrm{SiN}x}},$$

where l stands for the total circle length and w stands for the conductor width. ε_SiNx_ and t_SiNx_ denote the permittivity and thickness of the SiNx substrate, respectively. Similarly, the permittivity and thickness of the GaAs substrate can be written as R_sub_ and C_sub_ [34,35].

(5) $$R_{\mathrm{sub}}=\frac{2}{\mathrm{lw}G_{0}},\;$$

(6) $${\;C}_{\mathrm{sub}}=\frac{1}{2}\mathrm{lw}C_{0},$$

where G_0_ and C_0_ represent the conductance and the capacitance of the GaAs substrate. Therefore, the main properties and parameters can be derived as above. Meanwhile, the Debye equation is used to explore the relationship between the complex permittivity (ε = ε′ − jε″) of the environment and the frequency and amplitude of the microwave biosensor, which can be expressed as follows:

(7) $$\left[\begin{array}{c} ∆f_{0}\\ ∆\left|S_{11}\right| \end{array}\right]=\left[\begin{array}{cc} m_{11} & m_{12}\\ m_{21} & m_{22} \end{array}\right]\left[\begin{array}{c} ∆\varepsilon^{\prime }\\ ∆\varepsilon^{\prime \prime } \end{array}\right].$$

$∆\varepsilon^{\prime }=∆\varepsilon_{s}^{\prime }-∆\varepsilon_{r}^{\prime }$, $∆\varepsilon^{\prime \prime }=∆\varepsilon_{s}^{\prime \prime }-∆\varepsilon_{r}^{\prime \prime }$, $∆f_{0}=f_{s}-f_{r}$, and $∆\left|S_{11}\right|={\left|S_{11}\right|}_{s}-{\left|S_{11}\right|}_{r}$ are the differences between the sample (with subscript s) and the reference (with subscript r) values. The unknown coefficients (m_ij_, i, j = 1, 2) of the fitting matrix can be calculated from the approximated values of the complex permittivity and the RF parameters by applying the least-square method [36]. From the equation, it can be deduced that the variation in environmental dielectric constant, caused by yeast growth, results in changes to the biosensor’s resonant frequency and amplitude.

**Figure 2:**
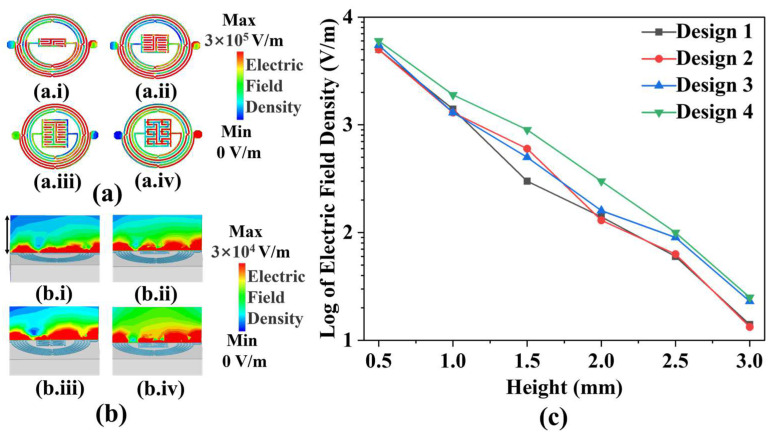
Performance of different design of biosensors: (**a**) different electric field density of (**a.i**) Design 1, (**a.ii**) Design 2, (**a.iii**) Design 3, and (**a.iv**) Design 4; (**b**) different electric field density of (**b.i**) Design 1, (**b.ii**) Design 2, (**b.iii**) Design 3, and (**b.iv**) Design 4 at the height of 0.4 mm; and (**c**) the variation of electric field density with height for Design 1 to Design 4.

Figure 2a depicts the shapes and electric field distributions of IPD-based biosensor designs. Design 1 to Design 4 comprises two closed interdigital capacitors, with the number of metal wires increasing from 4 to 12. Additionally, Design 3 is based on Design 4, which places 12 air bridges on the capacitor section. As can be observed in the figure, the electric field intensity improves as the number of wires increases. While maintaining the same number of wires and structural shape, the capacitor with air bridges exhibits a higher electric field intensity in its vicinity when compared to that without air bridges. Furthermore, the experiment reveals changes in yeast growth above the medium, necessitating a more robust propagation of microwave signals from the biosensor towards the top. Figure 2b illustrates the distribution of electric field density from Design 1 to Design 4 at a height of 0.4 mm. The electric field strength density is obtained by implementing a structural model in a High-Frequency Structure Simulator (HFSS v18.0) and performing finite element method simulations. It is apparent that Design 4 emits the highest electric field density upwards. Since the height of the added medium is 3 mm, in order to further analyze the variation of the electric field density radiated by the biosensors with height, Figure 2c shows the variation of the electric field density with height in the range of 0.5 mm to 3 mm, respectively, for Design 1 to Design 4. Although the electric field density radiated by the biosensor shows a decreasing trend with increasing height, Design 4 still has the highest electric field density among the four designs with 25 V/m at the height where the yeast grows in the experiment. Therefore, Design 4 was selected as the biosensor for the experiment.

## 3. Experiment and Discussion

### 3.1. Biological Sample Preparation

The solid medium required for yeast growth is YPAD medium, which is configured as follows: 0.4 g of adenine sulphate, 10 g of yeast extract, 20 g of peptone, and 20 g of agar are dissolved in 900 mL of DI water. In this step, the yeast extract provides a source of nitrogen and growth factors required for the growth of the yeast, while adenine sulphate is added to promote the growth of the yeast as a growth regulator. In addition, peptone provides a source of nitrogen, which is involved in the composition of microbial cells and nitrogen-containing metabolites, while agar acts as a solidifying agent to convert the medium to a solid state. The solution is autoclaved at 121 °C for 15 min for complete elimination of these mixed bacteria, ensuring the sterility of the culture medium. After cooling, 20 g of glucose and 100 mL of deionized water are added, and the solution is sterilized to provide essential carbon sources and energy materials for growth. Prior to use, the entire medium is maintained in a liquid state through heating in a microwave, facilitating uniform application onto the biosensor surface. To ensure experimental consistency, aliquots of 1200 µL and 540 µL of the prepared medium are dispensed into petri dishes for the preliminary and main experiments, respectively, ensuring a consistent medium thickness of 3 mm. 

The yeast used in the experiment is *Pichia pastoris* (strain GS115), which is prepared as follows: 1 µL of yeast (OD600 = 1.306, with a concentration of 2 × 10^7^ cells/mL) is added dropwise to a petri dish to which medium has been added beforehand and incubated continuously until the yeast growth enters plateau phase. A small amount of yeast is selected from the finished yeast colony and configured as a stock solution (yeast OD600 = 1.0, with a concentration of 1.5 × 10^7^ cells/mL) and 1 µL is added dropwise to the capacitive biosensor and the microwave biosensor, respectively, ensuring complete coverage of the sensitive area of the biosensor. Finally, the petri dishes are covered to ensure a sterile environment throughout the growth measurement experiment and to minimize water evaporation from the medium. All of these operations are carried out in a sterile environment.

### 3.2. Biosensors Used for Detection

**Figure 3:**
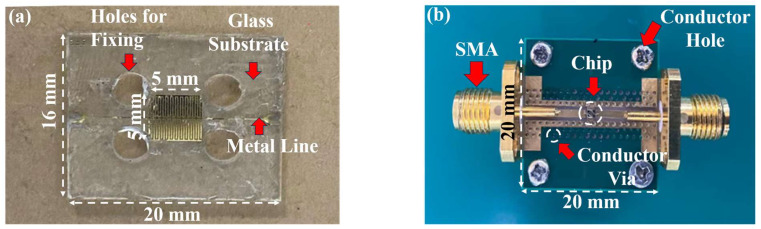
Proposed biosensors: (**a**) capacitive biosensor and (**b**) microwave biosensor.

Designed microwave biosensors as well as capacitive biosensors for microbial detection are shown in Figure 3. Figure 3a shows the capacitive device part of the biosensor, consisting of a thin copper layer forming the shape of a forked finger on a glass substrate with metal wires extending on both sides to match the impedance, where the central capacitor section has a dimension of about 5 × 5 mm^2^. Additionally, four holes are placed around the center capacitance area for device fixation. Figure 3b shows the proposed microwave biosensor. The biosensor, which comprises a microresonator with a 50 Ω impedance matching port and the SMA connector at both ends, is fixed to an aluminum block to ensure its connection to the ground. The capacitive biosensor is low-cost and simple in structure. The microwave biosensor operates at a high operating frequency for better signal penetration and has more microwave parameters to respond to environmental changes caused by microbial growth. Therefore, a feasibility experiment is first conducted on yeast growth using capacitive biosensors, and then a microwave biosensor is used for further measurements.

### 3.3. Pre-Experimental Environments and Capacitive Biosensor

**Figure 4:**
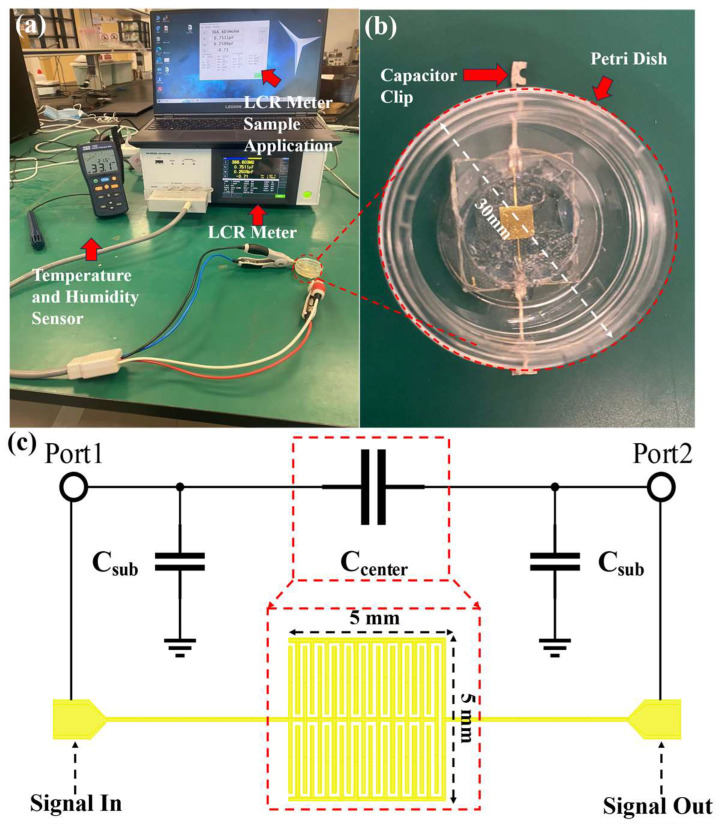
Experimental environment for pre-experiment and used capacitive biosensor: (**a**) experimental environment setup; (**b**) bonded petri dish of capacitive biosensor; and (**c**) structure and equivalent circuits of capacitive biosensors.

Figure 4a delineates the setup for the preliminary experimental environment. An LCR meter (KIOKI, IM3536) along with an LCR Sample Application are employed to continuously monitor the electrical parameters of the biosensor. A Temperature and Humidity Sensor (TES, 1364) tracks the ambient conditions, as detailed in Figure 4b. The biosensor configuration includes a capacitive device attached to a petri dish. To promote yeast growth on the biosensor, a petri dish with a diameter of 30 mm is affixed to the surface of the capacitor, as depicted in Figure 4c. A circular notch, 20 mm in diameter, is cut from the bottom of the petri dish, which is approximately 1 mm thick. This modification facilitates direct contact between the culture medium and the central capacitive section of the capacitor, minimizing potential electrical signal loss through the glass bottom of the petri dish. Capacitor clips are secured to the metal wires at both biosensor ends, enabling the LCR meter to accurately measure the biological electrical parameters of the biosensor. Figure 4c illustrates both the structure and equivalent circuits of capacitive biosensors, where C_center_ stands for the capacitance between metal wires and materials and C_sub_ represents the capacitance between metal wires and ground, which can be expressed as follows:

(8) $$C_{\mathrm{sub}}=\frac{\mathrm{wl}\varepsilon_{0}\varepsilon_{\mathrm{sub}}}{d}\;(F),$$

(9) $$C_{\mathrm{center}}=\frac{\varepsilon_{0}\varepsilon_{m}wd}{a}\;(F).$$

In the formula, the dimensions of the metal wires are denoted by w, l, and d for width, length, and thickness, respectively. The spacing between the wires is represented by a. The dielectric constant of free space and glass substrate are denoted by ε_0_ and ε_sub_, respectively. Yeast growth phase changes the dielectric constant ε_m_ in the environment, which alters the value of C_center_ and eventually results in the change in the total capacitance of the biosensor. The units of w, l, d, and a are all in meters, while the unit of ε_0_ is in F/m. As relative permittivity, ε_sub_ and ε_m_ have no units.

### 3.4. Pre-Experimental Results and Analysis

**Figure 5:**
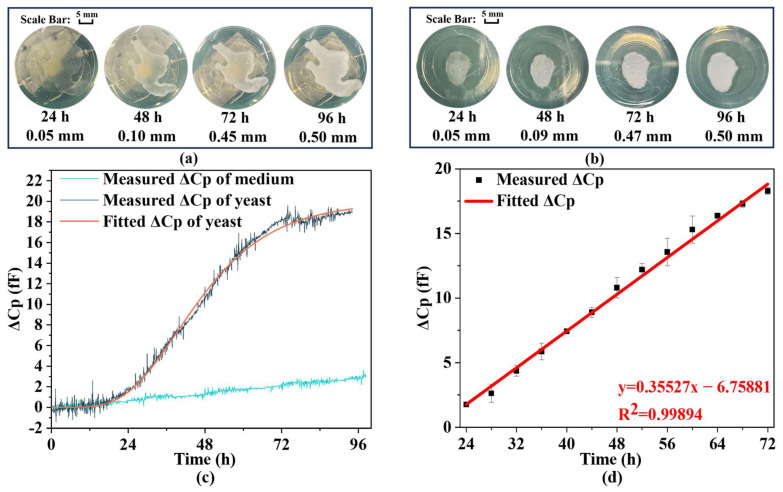
Record of growth of yeast: Growth situation of yeast on (**a**) biosensor and (**b**) petri dish with scale bar. (**c**) Measured ΔCp for 1 µL of yeast growth and the fitted Gompertz model curve at constant time intervals. (**d**) ΔCp measurements of yeasts every 4 h during the growth period with linear fit results with error bar. Note: error bars generated by fitting multiple measurement data using standard deviation (SD < 3.8%).

The biosensor is loaded with 1200 µL of the preconfigured medium to maintain a medium height of 3 mm. Measurements are continuously recorded using an LCR meter at a temperature of 20 ± 0.5 °C and an operating frequency of 1 MHz, with data captured every 5 min. Small changes in temperature do not affect the measurement results. The final dataset is recorded and plotted for analysis. To mitigate the influence of extraneous variables, the experiment incorporates two control groups. Control Group 1 involves loading an identical volume of medium into the same type of biosensor and connecting it to the LCR meter for continuous measurements to assess the effect of medium evaporation on the data. Control Group 2 adds the same quantity of yeast to a blank petri dish with an equivalent medium height, aiming to determine the yeast’s growth viability on the biosensor substrate. Figure 5a,b illustrate the yeast growth on the capacitive biosensor element and in the petri dish, respectively, indicating no significant difference in growth rates between the two environments, thereby confirming that the capacitive biosensor does not affect yeast proliferation. Figure 5c presents the results from the continuous measurements of the biosensor and Control Group 1, where parallel capacitance (Cp) is employed to describe the biosensor’s capacitance value, given its impedance exceeds 10 kΩ throughout the measurement period. After 100 h of continuous measurement, the capacitance of the biosensor with the yeast culture exhibits a decrease of 18 (fF).

To fit the obtained capacitance variation data, the Gompertz model is employed, which is commonly used to describe the growth of plants, animals, and microorganisms. The expression for the Gompertz model type I is as follows:

(10) $$y\left(t\right)=A\;\times \exp\left(-\exp\left(-K_{G}\times \;\left(t\;-{\;T}_{i}\right)\right)\right)\;(\mathrm{pF})$$

The expected variation in resonant amplitude as a function of time t is represented by y(t). A stands for the upper asymptote (adult value), K_G_ is the growth rate coefficient (which affects the slope), and T_i_ represents the time at inflection [37]. Table 1 displays fitted Gompertz growth model parameters and their uncertainties. 

The growth curve after fitting is as follows:

(11) $$y\left(t\right)=19.9229\;\times \exp\left(-\exp\left(-0.062\;\times \left(t-39.3111\right)\right)\right)\;(\mathrm{pF})$$

The data correlate strongly with the Gompertz model (R^2^ = 0.9961), accurately reflecting yeast growth dynamics. Furthermore, Figure 5c demonstrates a capacitance change in the culture medium, with a decrease of approximately 3 fF over 100 h, distinct from the capacitance recorded on the biosensor where the yeast is placed. This observation confirms that medium evaporation does not influence the measurements of yeast growth. Figure 5d shows a continuous change in capacitance (Cp) from 24 to 72 h, indicating a nearly linear relationship. These results suggest that changes in electrical parameters due to yeast proliferation can be translated into alterations in biosensor Cp, which can then be utilized as electrical signal outputs. These outputs may be connected to an external circuit for real-time monitoring of yeast growth. To explore this linear relationship further, capacitance measurements are taken every 4 h, with additional recordings 15 min before and after each time point. Given the stability of the yeast population during these 30 min, these measurements are considered equivalent, thereby enhancing the reliability and accuracy of the results. Figure 5d presents both the collected data throughout the growth cycle and the linear regression analysis. The change in Cp over time can be expressed as follows:

(12) $$Y\left(t\right)=0.35527t-6.75881\;\left(R^{2}=0.9989\right).$$

Y(t) represents the Cp change and t represents the time of growth. In conclusion, the peripheral circuitry can be employed to display and output real-time data on yeast growth throughout its proliferation phase. This study has established that the electrical properties of yeast are indeed modulated by its growth, while the experimental outcomes are negligibly affected by medium evaporation or device-related influences. For subsequent research, it is advisable to use an IPD-based biosensor made with higher sensitivity and smaller size to continuously measure the growth of yeast, which operates in microwave frequency bands with high penetration capability.

### 3.5. Experimental Environment and IPD-Based Biosensor

**Figure 6:**
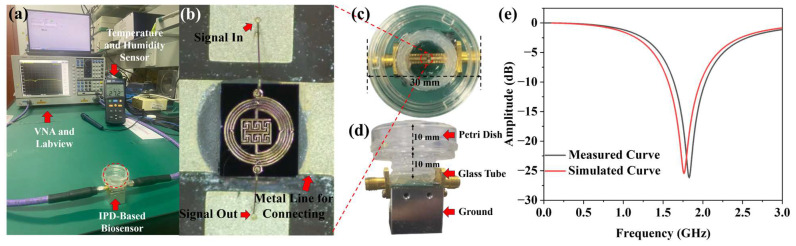
Experimental environment and used biosensor: (**a**) experimental environment setup; (**b**) microscope photo of IPD-based biosensor; (**c**) top view and (**d**) side view of the final test fixture; and (**e**) measured and simulated S11 parameters of biosensor.

Figure 6a illustrates the experimental environment for continuous IPD-based biosensor measurement. Yeast growth is continuously monitored using a Vector Network Analyzer (VNA, Ceyear, 3656B, Qingdao, China) and LabVIEW 2018 for data acquisition. The ambient temperature is monitored using a Temperature and Humidity Sensor. Figure 6b depicts a microscopic image of the central microresonator part. Two jumper wires are led out to connect the microresonator to the metal wires at both ends. In order for yeast to grow on the biosensor, a petri dish is bonded to it to create a suitable growth environment. Due to the size limitations of the SMA, a glass tube with a diameter of 15 mm and a height of 10 mm is glued at the bottom of the petri dish. The modified petri dish is then glued to the biosensor to ensure yeast growth during the experiment. Figure 6c,d illustrate the final fixture. Figure 6e depicts the S11 parameters of the biosensor, which are measured using VNA and compared with the software simulation results. The resonant frequency of the biosensor in the software simulation is 1.77 GHz with an amplitude of −25.34 dB. In the actual measurement, the resonant frequency is found to be 1.84 GHz with an amplitude of −26.23 dB. There is minimal discrepancy between the actual measurement and the simulation result which may be due to fabrication tolerance, a wire bonding effect, and connection orientation of the connectors soldered to the device [38].

## 4. Experimental Results and Analysis

**Figure 7:**
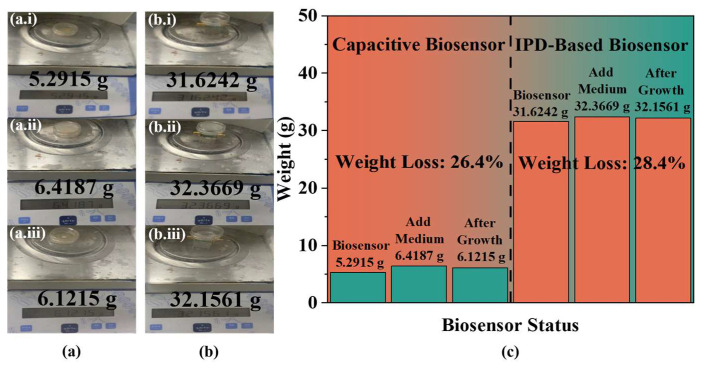
Weight of the biosensor at different stages: (**a**) weight of capacitive biosensor (**a.i**) before adding medium, (**a.ii**) after adding medium, and (**a.iii**) after 100 h of yeast growth; (**b**) weight of IPD-based biosensor (**b.i**) before adding medium, (**b.ii**) after adding medium, and (**b.iii**) after 100 h of yeast growth; and (**c**) weight changes and percentage of weight loss summarized in a bar chart.

To ensure accurate results, the height of the medium is maintained at 3 mm by adding 540 µL of culture medium to the biosensor, followed by the addition of 1 µL of yeast at the same concentration for analysis. Measurements are taken continuously at a controlled temperature of 20 ± 0.5 °C using VNA at a frequency of 1.84 GHz, with data recorded every 5 min through LabVIEW 2018 software. To mitigate the effects of evaporation on the experimental outcomes, the biosensor’s weight is measured before and after the addition of the medium and at the end of the growth cycle, as shown in Figure 7a,b. The results, depicted in Figure 7c through a bar chart, indicate that there is no significant change in the overall weight of the biosensors before and after the growth period. Further analysis compared the reduction in medium mass during and prior to the experiment, showing a decrease of 26.4% and 28.4% in capacitive and IPD-based biosensors, respectively, after a full growth cycle. These findings suggest that the weight reduction is proportionally similar across the same growth period, confirming that evaporative loss does not significantly influence the experimental results. Therefore, the inclusion of a control group specifically to account for evaporation is deemed unnecessary.

The growth of yeast is continuously monitored using an IPD-based biosensor with a measurement period of 100 h. In addition, the amplitude in the S11 curve is selected to characterize the results [39], with the variation of the amplitude over the measurement period. The amplitude change is primarily influenced by the loss angle tangent of the environment, which is associated with conductivity and enables the detection and monitoring of microbial growth. During the growth of yeast, the loss angle tangent of the environment undergoes continuous changes due to the vertical growth of yeast, resulting in fluctuations in the amplitude of the biosensor. Concurrently, the curve is fitted with the Gompertz model type I, as previously described in the preceding section. The resulting growth curve after fitting is as follows:

(13) $$y\left(t\right)=0.2658\;\times \exp\left(-\exp\left(-\;0.0398\;\times \left(t-45.4135\right)\right)\right).$$

**Figure 8:**
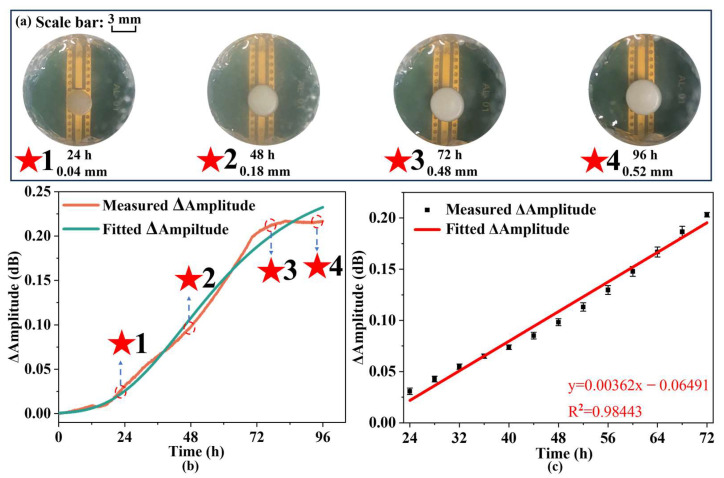
Record of experimental data and growth of yeast: (**a**) growth situation of yeast on biosensor with scale bar; (**b**) measured ΔAmplitude for 1 µL of yeast growth and the fitted Gompertz model curve at constant time intervals; and (**c**) measured ΔAmplitude of yeast every 4 h during the growth period with linear fit results with error bar. Note: error bars generated by fitting multiple measurement data using standard deviation (SD < 3.8%).

Table 2 displays fitted Gompertz growth model parameters and their uncertainties. Based on the good fit (R^2^ = 0.9917), the measured growth curves can be considered to be consistent with the growth pattern. Figure 8a illustrates the growth dynamics of yeast on the biosensor over various time intervals, showing a pronounced growth phase between 24 and 72 h, followed by a marked decline or stabilization after 72 h. This pattern aligns well with the biosensor-based measurements, indicating that the dielectric constant of the environment is altered during yeast proliferation in a solid culture medium. The biosensor, operating in the microwave frequency band, effectively captures these changes through amplitude variations, thereby facilitating real-time and continuous monitoring of yeast growth. Meanwhile, comparing the measurement results of the IPD-based biosensor with those of the capacitive biosensor for yeast, it is evident that the IPD-based biosensor provides more stable data over short periods, thus reducing measurement errors compared to the capacitive biosensor. Furthermore, the amplitude changes at specific intervals, every 4 h as well as 15 min before and after each interval, are continuously recorded. Figure 8b shows the data collected throughout the growth cycle alongside the linear fitting analysis. The change in amplitude over time can be expressed as follows:

(14) $$Y\left(t\right)=0.00362t-0.06491\;\left(R^{2}=0.9844\right).$$

Y(t) represents the amplitude change and t represents the time of growth. Therefore, it is possible to use IPD-based biosensors with peripheral circuits to monitor yeast population changes during the growth period.

**Figure 9:**
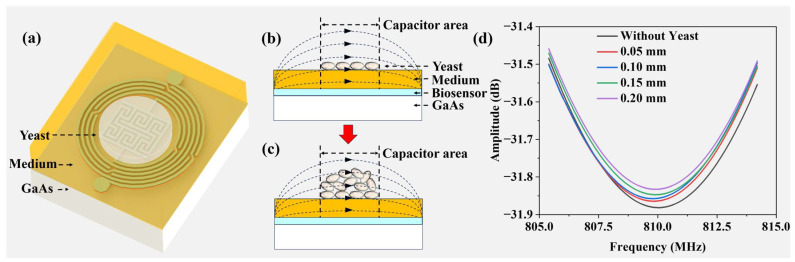
Mechanism diagrams for real-time monitoring of yeast growth: (**a**) 3D view of yeast growth on the biosensor; side view of yeast (**b**) before and (**c**) after growth; and (**d**) S11 parameters for yeast colony heights of 0.05, 0.10, 0.15, 0.20 mm, and without yeast colony on medium in simulation.

Figure 9a illustrates the three-dimensional yeast growth environment, where a 3 mm solid medium is situated atop the microwave biosensor, supporting the growth of the yeast colony. Figure 9b,c depict side views of the yeast growth process, noting that the height of the solid medium remains constant as the agar does not undergo evaporation. Throughout the growth process, the height of the yeast colony increases, whereas the base area remains unchanged. Correspondingly, the electrical properties of the yeast colony evolve with the increasing biomass, resulting in an expansion of the colony-induced electric field lines. To simulate yeast growth, HFSS v18.0 software is employed, using a rectangular model with a relative dielectric constant of 85, a loss tangent of 0.002, and a height of 3 mm, placed over the IPD-based biosensor as the culture medium. A cylindrical model, slightly exceeding the sensitive area of the IPD-based biosensor, is positioned on the surface of the rectangle to facilitate yeast growth. Figure 9d presents the frequency response of the biosensor within the range of 805 MHz to 815 MHz as the height of the cylinder is adjusted sequentially to 0 mm, 0.05 mm, 0.1 mm, 0.15 mm, and 0.20 mm, maintaining the dielectric constant of the yeast. This figure illustrates variations in the height of the yeast on the culture medium. The resonant frequency of the biosensor remains relatively stable, while the resonant amplitude reflects changes in the colony height. Throughout the yeast growth process, the amplitude of the biosensor varies, facilitating real-time monitoring of the growth. An objective analysis of the experimental results corroborates this observation.

Table 3 compares the performance of various biosensors designed for monitoring microbial growth. Contemporary research in microbiological measurements predominantly focuses on *E. coli*, with growth assays conducted in both solid and liquid media. Given that *E. coli* is a bacterial species and yeast is a fungal organism, their inherent structural differences may influence the outcomes of measurements. Despite this, current methodologies for real-time monitoring of yeast growth are primarily centered on liquid media, and there is a notable scarcity of studies investigating yeast growth on solid media. This research introduces a novel microwave biosensor capable of monitoring yeast growth on solid media. This IPD-based biosensor distinguishes itself from others by its compact size and high degree of integration which provides it the potential to match complete biosensor systems.

## 5. Conclusions

In this study, we propose a contactless microwave biosensor based on IPD technology and use yeast as model organism to demonstrate the capabilities of the biosensor system. The feasibility of the experiment is verified using a capacitive biosensor, and the microwave biosensor is used to detect yeast growth at an operating frequency of 1.84 GHz. The biosensors’ measurement results are consistent with those of microbial growth modeling, indicating their potential for real-time microbial monitoring. Of the two biosensors, the capacitive biosensor has a higher linearity of fit to the obtained data, indicating that the capacitive biosensor is more suitable for connecting matching circuits and creating complete measurement systems. Meanwhile, measurement data of the microwave biosensor are more stable in a short period of time, which proves that it is more promising for the exact detection of a small amount of samples in a short term. Additionally, the biosensor exhibits good linearity of signal changes during the growth phase, proving its suitability for integration into peripheral circuits to create a complete sensing system for real-time monitoring of microbial growth. In the future, further work, such as detecting the formation of biofilms, could be realized with the biosensor designed in this article which is of potential help in medical and other fields.

## Figures and Tables

**Table 1:** Fitted Gompertz growth model parameters and uncertainties for yeast measured via capacitive biosensor.

Model	$y(t)=A\;\times \exp\left(-\exp\left(-K_{G}\times \;(t\;-{\;T}_{i})\right)\right)$
A	19.9229 ± 0.05258
K_G_	0.062 ± 0.00048
T_i_	39.3111 ± 0.22076
R^2^	0.9961

**Table 2:** Fitted Gompertz growth model parameters and uncertainties for yeast measured via microwave biosensor.

Model	$y\left(t\right)=A\;\times \exp\left(-\exp\left(-K_{G}\times \;\left(t\;-{\;T}_{i}\right)\right)\right)$
A	0.2658 ± 0.00172
K_G_	0.0398 ± 0.00046
T_i_	45.4135 ± 0.22076
R^2^	0.9917

**Table 3:** Performance of different microbial growth monitoring biosensors.

Reference and Structure	Microbial Species and Environment	Maximum Signal Change	Size of Biosensor	Advantages
[29] LC Resonator	*E. coli* Solid Medium	0.025 dB 0.3 MHz	20 × 10 mm^2^	High quality factor
[39] LC Resonator	*E. coli* Solid Medium	0.18 dB 0.09 dB	20 × 12 mm^2^	High linearity
[40] Capacitive Sensor	*E. coli* Liquid Medium	128 fF	2 mm^2^	Portable
[41] Light Intensity-modulated Biosensor	*E. coli*, Yeast Liquid Medium	4 V	Not Given cm^2^	Low cost and complexity
[42] Electrochemical Dynamics Biosensor	Yeast Liquid Medium	100 Om	Not Given cm^2^	High anti-interference capability
**This Work** **IPD-Based Biosensor**	**Yeast** **Solid Medium**	**0.21 dB**	**980 × 800** **μm^2^**	**Highly integrated and small in size**

## Data Availability

Data are contained within the article.
